# A novel insight of sentinel lymph node concept based on 1-3 positive nodes in patients with pT1-2 gastric cancer

**DOI:** 10.1186/1471-2407-11-18

**Published:** 2011-01-17

**Authors:** Baojun Huang, Zhenning Wang, Zhe Sun, Bo Zhao, Huimian Xu

**Affiliations:** 1 Department of Surgical Oncology, the First Hospital of China Medical University, Shenyang, 110001, PR China; 2 Department of Oncological Sciences, Mount Sinai School of Medicine, New York, NY 10029, USA

## Abstract

**Background:**

Sentinel node (SN) biopsy has been practiced in gastric cancer in recent years, and many studies focused on the distribution of solitary lymph node metastasis (SLM) to assess the pattern of SN. In fact, there is usually more than one SN existing in gastric cancer. The distribution of SNs needs to be further re-evaluated.

**Methods:**

A total of 289 patients in pT1-2 stage with 1-3 positive nodes confined to same compartment were included in this study with informed consents. The primary lesion was solitary (≤ 5.0 cm in diameter) and D2 or D3 lymph node dissection had been performed. The location of metastatic lymph nodes was analyzed retrospectively.

**Results:**

Most positive nodes occurred in N1 compartment, with frequency of 79.6% to 85.7% based on site of tumor. In the lower third of stomach, no. 6 was the most common metastatic site and no. 3 was the second; the order was reversed for SLM. With increasing depth of tumor invasion, a progressively augmented nodal involvement was shown. Nearly a half appeared transverse metastasis when the tumor located at the lesser or greater curvature. Among skip metastasis, no. 7, 8a, 9 and 11p were the most common metastatic sites and the prognosis was as similar as that of patients with N1 involved only.

**Conclusions:**

The 1-3 positive nodes in the same compartment should be possible SNs, and most of which are restricted in N1 in pT1-2 gastric cancer. Transversal and 2 stations lymph node metastasis are common.

## Background

Although the incidence of gastric cancer is declining, it remains the second leading cause of cancer related mortality worldwide [1-3]. Lymph node status is one of the crucial important prognostic factors, and gastrectomy with D2 or D3 lymphadenectomy is still considered as the only treatment offering hope of a cure for gastric cancer. However, the incidence of nodal involvement in gastric cancer is significantly different according to the depth of tumor invasion (T1-T4) [4-7]. Uniform application of this highly invasive procedure would increase morbidity and reduce the quality of life after surgery, especially for those with early stage cancers. However, the optimal strategy of lymphadenectomy for gastric cancer is still under debate.

Sentinel node (SN) is defined as the first lymph node which receives lymphatic drainage from the primary tumor. Sentinel node biopsy (SNB) has been widely applicated as an alternate treatment to maintain the quality of life for cT1-2 gastric cancer patients in recent years. However, this technique is still unsatisfactory for clinical application due to its high heterogeneity in sensitivity, specificity and accuracy (from 61.1% to 100%) [8-12]. There are several potential explanations for these results: 1) different examining methods which need to be standardized; 2) limited sample size of participants and sentinel nodes examined; 3) patients in late stage included occasionally; 4) multidirectional and complicated lymphatic flow from stomach. Resolving the above problems could improve the accuracy of SNB practice in gastric cancer.

Up to now, many studies have investigated the localization and distribution of solitary lymph node metastasis (SLM) in order to provide some useful information for SN concept in gastric cancer, which may offset the insufficiency of study sample size in SNB studies [13-16]. However, the lymphatic drainage of the stomach is considerably more complex than that of ectodermal organs like breast and skin. The multidirectional and complicated lymphatic flow from stomach results in more than one node, which should be considered as SN for gastric cancer. Furthermore, many investigations have showed that the number of SN per patient is 1-12 with an average of 3 [8,17-19]. Consequently, using SLM to assess the distribution of SNs in gastric cancer may leave out some important information, thus it might be more appropriate to practice the SN concept based on more than one metastatic lymph node.

In the light of these considerations, the aim of the present study was to assess the distribution of 1-3 positive nodes in pT1-2 gastric cancer patients. This would provide some new information for the concept of SNs in gastric cancer, especially in early stage of tumor.

## Methods

A retrospective analysis of clinicopathologic data for gastric cancer patients from a prospectively collected gastric cancer database from February 1980 to November 2006, at the Department of Surgical Oncology, First Affiliated Hospital, China Medical University, was performed. The criteria for inclusion in this study were as follows: (1) primary lesion was solitary (≤ 5.0 cm in diameter) and limited to one part of the stomach; (2) patients were in pT1-2 stage according to the 6^th ^UICC/TNM classification; (3) D2 or D3 lymph node dissection had been performed; (4) patients had 1-3 metastatic lymph nodes which restricted to the same compartment according to JCGC; (5) the number of examined lymph node was more than 10; (6) all the patients clinically staged as no macroscopic serosal invasion (cT1-2) and node negative (cN0) before or during surgery. A total of 297 patients with gastric cancer were included. At the end of follow-up in December 2008, 4 patients died in the postoperative period and 4 patients were lost, with the follow-up rate of 97.3%. The median and mean follow-up period was 45.0 and 68.8 months (3-342 months), respectively. Overall, 289 patients with gastric cancer were enrolled into this study with their informed consents.

We first retrospectively investigated the localization and distribution of 1-3 positive nodes confined to the same compartment according to Japanese Classification of Gastric Carcinoma (JCGC), which were regarded as possible sentinel lymph nodes, and then compared those with solitary lymph node metastasis (SLM). The differences were identified between the two groups. Then the clinical and pathological features were analyzed for patients with transversal and two stations metastasis.

The study protocol was approved by the Ethics Committee of China Medical University.

### Statistical analysis

Data were analyzed using the SPSS statistical software (SPSS, Chicago, IL). The difference of nodes distribution between SLM and 1-3 positive nodes was tested by the chi-square test or Fisher's exact probability test. The univariate analysis was used by the chi-square test for categorical variables and unpaired t-test for continuous variables between groups. The multivariate analysis was used binary logistic regression. The survival analysis was used by Kaplan-Meier estimation and log-rank test. *P *< 0.05 was considered to be statistically significant.

## Results

### Clinicopathologic features

Among the patients, the age (mean ± SD) was 58.9 ± 10.4 years (ranging from 29 to 84 years). More men than women (224 men versus 65 women) participated in the study. Carcinomas were located in the lower third of stomach (L) in 207 patients, middle third (M) in 28 patients and upper third (U) in 54 patients. Tumors were located in the lesser curvature in 131 patients and in the greater curvature in 63 patients, respectively. Distal-gastrectomy was executed in 223 patients, proximal-gastrectomy in 47 patients and total-gastrectomy in 19 patients. Lymphadenectomy was executed based on the Japanese Classification of Gastric Carcinoma (JCGC) [20]. D2 lymph node dissection was performed in 212 patients, and D3 in 77 patients. The number of lymph node retrieved ranged from 10 to 55 with an average of 20.9 ± 9.9 (mean ± SD). Among them 10-14 retrieved nodes were in 97 patients and ≥ 15 retrieved nodes in 192 patients, respectively.

According to the depth of tumor invasion, pT1 cancer was diagnosed in 28 patients (9.7%), with protruded type (I, IIa) in 3 patients (10.7%) and depressed type (IIc, III) in 25 patients (89.3%), respectively. The pT2a cancer was diagnosed in 92 patients (31.8%), and pT2b cancer in 169 patients (58.5%), with Borrmann I/II in 54 patients (20.7%) and Borrmann III/IV in 207 patients (79.3%) based on macroscopic type. The tumor diameter ranged from 0.5 to 5.0 cm with an average of 3.9 ± 1.1 cm (mean ± SD). Well and/or moderately differentiated tumor was found in 129 patients (44.6%), and poorly differentiated tumor in 160 patients (55.4%) according to the histology. Diffuse-type was found in 170 patients (58.8%), intestinal-type in 111 patients (38.4%), and mixed-type carcinoma in 8 patients (2.8%) based on Lauren classification. SLM was found in 173 patients, 2 positive nodes in 75 patients, and 3 positive nodes in 41 patients, respectively. The metastatic lymph node restricted to one station was in 235 patients and two stations in the same compartment in 54 patients. The N1 compartment nodes were involved in 237 patients and N2 (skip metastasis) in 52 patients in light of JCGC.

### Location and distribution of 1-3 metastatic lymph nodes in gastric cancer

Among 207 patients with lower-third tumor, 170 patients (82.1%) had lymph node metastasis in the perigastric nodes (N1) close to the primary tumor and no. 6/3 was the most common site. The other 37 patients (17.9%) were found in N2 without N1 involvement. Of 28 patients with middle-third tumor, 24 patients (85.7%) had lymph node metastasis in N1 and skip metastasis was found in 4 patients (14.3%). In 54 patients with upper-third tumor, 43 patients (79.6%) metastasized in N1, and skip metastasis occurred in 11 patients (20.4%). In N2 compartment, no. 11p and 12a were also involved apart from no. 7, 8a and 9. The detailed frequency of different station involved in N1 and N2 was displayed in table 1.

**Table 1:** Localization and distribution of 1-3 positive lymph nodes in 289 patients with gastric cancer

	Tumor Location		
**station**	**L (%)**	**M (%)**	**U (%)**

no.1	8 (3.9)	3 (10.7)	24 (44.4)
no.2	-	1 (3.6)	9 (16.7)
no.3	62 (30.0)	13 (46.4)	15 (27.8)
no.4d	37 (17.9)	6 (21.4)	4 (7.4)
no.5	29 (14.0)	5 (17.9)	0
no.6	75 (36.2)	2 (7.1)	0
no.7	12 (5.8)	3 (10.7)	6 (11.1)
no.8a	13 (6.3)	1 (3.6)	4 (7.4)
no.9	3 (1.4)	0	2 (3.7)
no.10	0	-	1 (1.9)
no.11p	3 (1.4)	0	2 (3.7)
no.12a	0	1 (3.6)	0
compartment (JCGC)			
N1	170 (82.1)	24 (85.7)	43 (79.6)
N2	37 (17.9)	4 (14.3)	11 (20.4)
number of station			
1	172 (83.1)	21 (75.0)	41 (75.9)
2	35 (16.9)	7 (25.0)	13 (24.1)

### Difference of location and distribution between SLM and 1-3 positive nodes in gastric cancer

With respect to the tumor in lower third of stomach, there was no significant difference in frequency and distribution of skip metastasis between SLM and 1-3 positive nodes, and the no. 7, 8a and 9 were the most common target stations. In N1 compartment, the frequency of no. 5 and no. 6 involved in patients with 1-3 positive nodes was higher than that in patients with SLM (*p *< 0.05). Furthermore, in patients with 1-3 positive nodes no. 6 was the most common metastatic site, and no. 3 was the second, this was reversed to that in patients with SLM. As to the tumor in middle and upper third of stomach, the location and distribution of metastatic node in N1 and N2 compartment was similar in patients with SLM and 1-3 positive nodes, there was no significant difference (*p *> 0.05) (Table 2).

**Table 2:** Difference between distribution of SLM and 1-3 positive nodes in gastric cancer

	Lower third (%)				Middle third (%)				Upper third (%)			
						
station	SLM	1-3	x	*p*	SLM	1-3	x	*p*	SLM	1-3	x	*p*
**no.1**	6.2	3.9	0.96	0.328	5.9	10.7	0.31	0.581	33.3	44.4	0.92	0.337
**no.2**	-	-	-	-	5.9	3.6	0.13	0.715	18.5	16.7	0.04	0.835
**no.3**	27.9	30	0.16	0.688	41.2	46.4	0.12	0.731	29.6	27.8	0.03	0.862
**no.4d**	15.5	17.9	0.32	0.573	17.6	21.4	0.10	0.758	7.4	7.4	0.00	1.000
**no.5**	6.9	14	3.92	0.048*	11.8	17.9	0.30	0.585	0	0.0	-	-
**no.6**	25.6	36.2	4.13	0.042*	0	7.1	1.27	0.260	0	0.0	-	-
**no.7**	7.8	5.8	0.50	0.481	11.8	10.7	0.01	0.913	7.4	11.1	0.28	0.598
**no.8a**	6.9	6.3	0.06	0.802	5.9	3.6	0.13	0.715	3.7	7.4	0.43	0.514
**no.9**	2.3	1.4	0.35	0.555	0	0.0	-	-	0	3.7	1.03	0.311
**compartment**												
**N1**	76.0	82.1	1.87	0.172	82.4	85.7	0.09	0.763	88.9	79.6	1.91	0.167
**N2**	24.0	17.9			17.6	14.3			11.1	20.4		

note: SLM refers to solitary lymph node metastasis; 1-3 refers to 1-3 positive nodes.

* indicates the difference is significant.

### Pattern of distribution of 1-3 metastatic lymph nodes in gastric cancer according to the depth of tumor invasion

Among the tumors of different depth of invasion (T1, T2a and T2b), the pattern of lymph node metastasis in N1 and N2 compartment was similar. With an increase of T parameter, a progressively augmented nodal involvement was showed in some stations. In lower third of stomach, no. 6 was the most common station, from 25% in T1 to 39% in T2a, and to 36.4% in T2b tumors. The no. 3 was the second common, from 25% in T1 to 26% in T2a, and to 33.6% in T2b tumors. The no. 1 and no. 9 was not involved in T1 tumors, but was involved in T2a and T2b tumors. In middle third of stomach, no. 3 was the most common station, 40% in T1, 37.5% in T2a and 53.3% in T2b tumors. In upper third of stomach, no. 1 and no. 3 was the common metastatic site, and the frequency of N2 involved was much higher in T2b than that in T2a and T1 tumors. The skip metastasis often occurred in no. 7, 8a, 9, 10, and 11p in T2b tumors, but it seldom occurred in T2a and T1 tumors (Table 3).

**Table 3:** Incidence of 1-3 positive lymph nodes in gastric cancer patients according to the depth of tumor invasion (%)

	Lower third			Middle third			Upper third		
	
station	T1	T2a	T2b	T1	T2a	T2b	T1	T2a	T2b
no.1	0	2 (2.6)	6 (5.5)	3 (60.0)	0	0	1 (33.3)	5 (71.4)	18 (40.9)
no.2	-	-	-	-	1 (12.5)	0	0	1 (14.3)	8 (18.2)
no.3	5 (25.0)	20 (26.0)	37 (33.6)	2 (40.0)	3 (37.5)	8 (53.3)	1 (33.3)	2 (28.6)	12 (27.3)
no.4d	6 (30.0)	15 (19.5)	16 (14.5)	0	1 (12.5)	5 (33.3)	0	0	4 (9.1)
no.5	2 (10.0)	12 (15.6)	15 (13.6)	0	2 (25.0)	3 (20.0)	0	0	0
no.6	5 (25.0)	30 (39.0)	40 (36.4)	0	1 (12.5)	1 (6.7)	0	0	0
no.7	2 (10.0)	5 (6.5)	5 (4.5)	0	2 (25.0)	1 (6.7)	1 (33.3)	0	6 (13.6)
no.8a	1 (5.0)	3 (3.9)	9 (8.2)	1 (20.0)	0	0	-	0	3 (6.8)
no.9	0	2 (2.6)	1 (0.9)	0	0	0	-	-	2 (4.5)
no.10	-	-	0	-	-	-	-	-	1 (2.3)
no.11p	-	1 (1.3)	2 (1.8)	-	0	0	-	0	2 (4.5)
no.12a	0	0	0	0	1 (12.5)	0	-	0	0
compartment (JCGC)									
N1	18 (90.0)	64 (83.1)	88 (80.0)	4 (80.0)	6 (75.0)	14 (93.3)	2 (66.7)	7 (100.0)	34 (77.3)
N2	2 (10.0)	13 (16.9)	22 (20.0)	1 (20.0)	2 (25.0)	1 (6.7)	1 (33.3)	-	10 (22.7)
number of station									
1	19 (95.0)	65 (84.4)	89 (80.9)	4 (80.0)	5 (62.5)	12 (80.0)	3 (100.0)	6 (85.7)	32 (72.7)
2	1 (5.0)	12 (15.6)	21 (19.1)	1 (20.0)	3 (37.5)	3 (20.0)	-	1 (14.3)	12 (27.3)

### Transversal and two stations metastasis with 1-3 metastatic lymph nodes in gastric cancer

A total of 32 (50.8%) in the 63 patients with tumor in the greater curvature side had transversal metastasis. There were 5 cases metastasis in no. 1 station and 4 cases in no. 3 station among 13 patients in the upper third of stomach, and 2 cases in no. 3 and 1 case in no. 5 among 5 patients in the middle, and 14 cases in no. 3 and 6 cases in no. 5 among 45 patients in the lower third of stomach. 54 (41.2%) in the 131 patients with tumor in the lesser curvature side had transversal metastasis. There were 5 cases in no. 2 among 22 patients in the upper third of stomach, and just 1 case in no. 6 among 11 patients in the middle, and 15 cases in no. 4 and 33 cases in no. 6 among 98 patients in the lower third of stomach.

With respect to patients with two stations involved, in 34 tumors located in the lower third of stomach, 11 (32.4%) patients metastasized simultaneously in no. 5/6. No. 3/4 and no. 3/6 were both involved in 6 (17.6%) and 7 (20.6%) patients, respectively. The rest patients metastasized in no. 4/5, no. 4/6 and no. 3/5. Of note, 2 cases metastasized in no. 7/8a without N1 involvement. One was proved as sm (pT1) and the other as mp (pT2a) pathologically. Among 7 patients in the middle, no. 3/4, no. 5/6 and no. 1/3 were common metastatic stations. In 13 patients located in the upper third of stomach, no. 1/3 were the most common target sites, metastasized in 4 (30.8%) patients. It was worth noting that 4 cases with two stations involved appeared in N2 compartment (no. 8/9, no. 9/11, no. 8/10 and no. 7/11 stations involved, respectively), which amounted to 1.1% in 44 patients of upper third of stomach with 1-3 positive nodes, and who were all proved as ss (pT2b) pathologically.

In order to find some associated factors with transversal and 2 stations node involved, the correlation between clinicopathological features and them was analyzed. However, there was no significant association between them (detailed in table 4). The most possible reason for the high frequency of transversal and 2 stations lymph node metastasis might be the multidirectional and complicated lymphatic flow from stomach.

**Table 4:** The correlation between clinicopathological factors and transversal and 2 stations lymph node metastasis

		transversal metastasis				2 station positive nodes			
		
factors		- (%)	+ (%)	**x**^**2**^	*P *value	- (%)	+ (%)	**x**^**2**^	*P *value
sex	male	88 (58.3)	63 (41.7)	1.878	0.171	182 (81.3)	42 (18.7)	0.003	0.958
	female	20 (46.5)	23 (53.5)			53 (81.5)	12 (18.5)		
age (year)	mean ± sd	58.4 ± 9.8	58.3 ± 11.5	0.005	0.942*	58.6 ± 10.2	59.5 ± 11.2	0.204	0.652*
tumor size(cm)	mean ± sd	3.6 ± 1.1	3.5 ± 1.3	0.195	0.659*	3.9 ± 1.2	4.0 ± 1.0	0.581	0.446*
lymphadenec-tomy	D2	81 (54.4)	68 (45.6)	0.445	0.505	168 (79.2)	44 (20.8)	2.243	0.134
	more than D2	27 (60.0)	18 (40.0)			67 (87.0)	10 (13.0)		
	L	75 (52.4)	68 (47.6)			173 (83.6)	34 (16.4)		
tumor site	M	12 (75.0)	4 (25.0)	3.29	0.193	21 (75.0)	7 (25.0)	2.463	0.292
	U	21 (60.0)	14 (40.0)			41 (75.9)	13 (24.1)		
depth of tumor invasion	T1	14 (58.3)	10 (41.7)			26 (92.9)	2 (7.1)		
	T2a	31 (49.2)	32 (50.8)	1.582	0.453	76 (82.6)	16 (17.4)	3.318	0.19
	T2b	63 (58.9)	44 (41.1)			133 (78.7)	36 (21.3)		
differentiation	well/moderately	47 (54.0)	40 (46.0)	0.173	0.677	102 (79.1)	27 (20.9)	0.773	0.379
	poorly	61 (57.0)	46 (43.0)			133 (83.1)	27 (16.9)		
macroscopic type	Borr. 1/2	22 (64.7)	12 (35.3)	1.523	0.217	47 (87.0)	7 (13.0)	2.068	0.15
	Borr. 3/4	72 (52.9)	64 (47.1)			162 (78.3)	45 (21.7)		
lauren type	intestenal	41 (55.4)	33 (44.6)			85 (76.6)	26 (23.4)		
	diffused	64 (56.1)	50 (43.9)	0.09	0.956	142 (83.5)	28 (16.5)	4.027	0.134
	mixed	3 (50.0)	3 (50.0)			8 (100.0)	0 (0.0)		
lymphatic/venous invasion	-	89 (55.3)	72 (44.7)	0.059	0.809	196 (80.7)	47 (19.3)	0.433	0.511
	+	19 (57.6)	14 (42.4)			39 (84.8)	7 (15.2)		

note: * the difference was tested by unpaired t test

### Influenced factors and survival analysis of skip metastasis

A total of 52 patients occurred skip metastasis. In order to find factors influencing skip metastasis, the correlation was assessed between skip metastasis and clinicopathologic factors which includes gender, age, tumor location, tumor size, macroscopic type, differentiation, Lauren classification, depth of tumor invasion and vessel involvement. As a result, no clinicopathologic factor was found to be associated with skip metastasis using univariate and multivariate analysis (data not shown).

**Figure 1:**
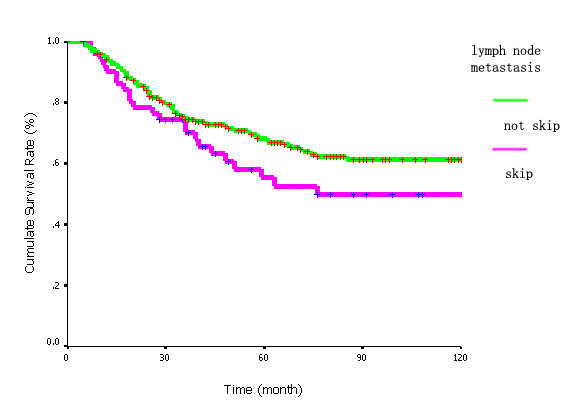
**Survival curves of patients with and without skip metastasis**. No significant difference was detected among patients with and without skip metastasis (x^2 ^= 0.168, *p *= 0.1951).

A little decrease of survival rate was showed in patients with skip metastasis. The 5-year, 10-year survival rate in patients with and without skip metastasis was 55.3%, 49.7% and 68.2%, 61.3%, respectively. However, no significant difference was detected between the two groups (x^2 ^= 0.168, *p *= 0.1951) using log rank test (figure 1), which meant the prognosis of patients with skip metastasis (N2) was as similar as that of patients with N1 involvement.

## Discussion

In order to accurately assess the sentinel lymph node distribution in gastric cancer, the criteria for inclusion in this study were defined very strictly. First, the primary tumor of 5.0 cm or less in diameter ensured that the lesion occupied only one part of the stomach, which could diminish the interactive effect from location overlap. Ichikura T et al. assessed the applicability of the sentinel node concept to gastric carcinoma based on 119 patients with a primary tumor ≤ 5.0 cm in diameter [21]. Yasuda K et al. defined a tumor measuring ≥ 5.0 cm as superficially spreading cancer of the stomach [22]. These literatures also support that 5.0 cm or less in diameter is suitable for SNs study in gastric cancer. Second, only patients with pT1-2 stage were selected and who were all clinically staged as cT_1/2_cN_0_, as these patients usually considered eligible for sentinel node trial. It was reported that the sensitivity decreased and false-negative rate increased with an increase of T stage in SNB [10,17]. Only pT1-2 stage inclusion avoided the bias due to the lymphatic obstruction in advanced gastric carcinoma in SNB study. Third, patients with 1-3 positive nodes only restricted in the same compartment based on JCGC classification were chosen. Isozaki H et al. reported that two patients with sentinel nodes in no. 4d also had another lymph node metastasis at the lesser curvature [17]. This phenomenon was also discovered by Osaka H et al. from micrometastasis level [23]. In their study, four patients with 2-5 blue-dyed nodes had micrometastasis in 2-3 nodes. Considering the number of SN per patient is 1-12 with an average of 3 and those mentioned above, it is more reasonable that 1-3 positive nodes in the same compartment are considered as the initial lymphatic drainage sites (SNs).

In this study, we valuated the 1-3 positive nodes distribution and compared those with SLM. Most positive nodes occurred in N1 compartment, with frequency from 79.6% to 85.7% based on different tumor site. The other 14.3% to 20.4% patients metastasized in N2 compartment directly without N1 involved. The no. 7, 8a, 9 and 11p stations were the most common sites. Our results were consistent with other reports, in which lymph node metastasis was distributed beyond the perigastric area in 12.6% to 29.0% of gastric cancer patients [15,16,21,24-26]. When compared with SLM, indeed some differences existed in node distribution between them. In patients located in the lower third of stomach with 1-3 positive nodes, the frequency of no. 5 and no. 6 infiltrated was higher than that in SLM, and no. 6 was the most common metastatic site, no. 3 was the second, the order was reversed for SLM. Among the cancers of other site, the location and distribution of positive nodes in N1 and N2 (skip metastasis) was similar. This is a novel insight about SNs distribution in gastric cancer that never reported before.

To know the distribution of the SNs contributes to choose more suitable lymphadenectomy. In the present study, transverse metastasis was quite common, amounted to 41.2% and 50.8% in the lesser and greater curvature, respectively. The rate is a little higher than previous reports [21,24]. The patients with more than one positive node included in this study maybe the main reason. It was also frequent that two stations were involved simultaneously, most of which occurred in the neighboring or opposite stations in the same compartment. Furthermore, with increasing the depth of tumor invasion, a progressively augmented nodal involvement was showed, including the number of stations involved and the frequency of some stations especially in N2 compartment. 4 patients in the upper third of stomach with two stations involved in N2 compartment were all proved as ss (pT2b) pathologically. All of above indicate that single lymph node dissection is not recommended, and the en-bloc dissection of lymphatic basins from the cancer should be performed to avoid the occurrence of false negative SLN, in the context of SLN biopsy, as the existence of high frequency of transversal and 2 stations lymph node metastasis.

Achieving an R0 resection is a critical step in obtaining local-regional control, but limitting the extent of lymphadenectomy is apt to expose some patients to the possibility of incomplete dissection. Skip metastasis was found in 14.3%-20.4% patients with 1-3 metastatic lymph nodes. When the influenced factors on skip metastasis were analyzed, none was found to be associated with it. This was also confirmed by Li C and Park SS [14,27]. Of note, the prognosis of patients with skip metastasis was as similar as that without skip metastasis after D2 or D3 lymphadenectomy. This means that in cases where metastasis first occurs in N2, the function of N2 in this situation is considered to be the same as N1. It suggests that we would achieve good surgical outcome if skip metastasis is found and dissected thoroughly. In order to obtain regional control, D2 lymphadenectomy is essential in patients with pT2 stage, as the higher occurrence of skip metastasis and higher frequency of some stations involved in N2 compartment in these patients.

## Conclusions

In a word, although the results from this study don't present the distribution of SN in gastric carcinoma directly, they could provide some valuable information for the use of SN concept in the treatment. We can conclude that 1-3 positive nodes in the same compartment should be possible SNs, and most of which are restricted in N1 in pT1-2 gastric cancer. Transversal and 2 stations lymph node metastasis are common.

## List of Abbreviations

SN: refers to sentinel node; SLM: refers to solitary lymph node metastasis; SNB: refers to sentinel node biopsy; sm: refers to submucosa based on depth of tumor invasion; mp: refers to muscularis propria based on depth of tumor invasion; ss: refers to subserosa based on depth of tumor invasion.

## Competing interests

The authors declare that they have no competing interests.

## Authors' contributions

HBJ and XHM conceived the study, analysed data, and drafted the manuscript and submitted the manuscript. WZN and SZ revised the manuscript critically for important intellectual content.

ZB conceived of the study and helped in drafting the manuscript. All authors read and approved the final manuscript.

## Pre-publication history

The pre-publication history for this paper can be accessed here:

http://www.biomedcentral.com/1471-2407/11/18/prepub
